# Advances in the knowledge and therapeutics of schizophrenia, major depression disorder, and bipolar disorder from human brain organoid research

**DOI:** 10.3389/fpsyt.2023.1178494

**Published:** 2023-07-12

**Authors:** Rosa Villanueva

**Affiliations:** Departamento de Psiquiatría y Salud Mental, Hospital Universitario La Paz, La Paz, Madrid, Spain

**Keywords:** modeling psychiatric disorders, induced pluripotent stem cells, tridimensional neural cultures, brain organoids, organ-on-chip, transdiagnostic psychiatry

## Abstract

Tridimensional cultures of human induced pluripotent cells (iPSCs) experimentally directed to neural differentiation, termed “brain organoids” are now employed as an *in vitro* assay that recapitulates early developmental stages of nervous tissue differentiation. Technical progress in culture methodology enabled the generation of regionally specialized organoids with structural and neurochemical characters of distinct encephalic regions. The technical process of organoid elaboration is undergoing progressively implementation, but current robustness of the assay has attracted the attention of psychiatric research to substitute/complement animal experimentation for analyzing the pathophysiology of psychiatric disorders. Numerous morphological, structural, molecular and functional insights of psychiatric disorders have been uncovered by comparing brain organoids made with iPSCs obtained from control healthy subjects and psychiatric patients. Brain organoids were also employed for analyzing the response to conventional treatments, to search for new drugs, and to anticipate the therapeutic response of individual patients in a personalized manner. In this review, we gather data obtained by studying cerebral organoids made from iPSCs of patients of the three most frequent serious psychiatric disorders: schizophrenia, major depression disorder, and bipolar disorder. Among the data obtained in these studies, we emphasize: (i) that the origin of these pathologies takes place in the stages of embryonic development; (ii) the existence of shared molecular pathogenic aspects among patients of the three distinct disorders; (iii) the occurrence of molecular differences between patients bearing the same disorder, and (iv) that functional alterations can be activated or aggravated by environmental signals in patients bearing genetic risk for these disorders.

## Introduction

Preclinical psychiatric research has attempted for decades to generate animal models that replicate mental disorders (1-3). The advances obtained in the study of the human genome, and the alterations detected in patients with different neuropsychiatric pathologies have contributed to consolidating the generation of genetically modified animal models to uncover pathogenic mechanisms of behavioral alterations. However, the structural complexity of the nervous system and human behavior compared to other mammals (4–8) together with the polygenic nature of the genome alterations detected in psychiatric disorders (9), limited the progress of psychiatry based on preclinical studies using animal models. It must be taken into account that rodents, used more frequently in biomedical research, from an evolutionary point of view are more than 90 million years older in their origin compared to humans and have enormous structural and functional differences (10).

From initial studies in the middle of the last century, modeling neural tissue differentiation by *in vitro* assays, constituted a promising tool to overcome limitations of animal models for the study neuropsychiatric disorders. The conventional 2D *in vitro* approaches enabled the study of morphological, molecular, and electrophysiological features of neurons and glia, providing remarkable insights in the mechanisms that regulate normal and abnormal neural tissue differentiation, as well as, for the identification of alterations induced in response to drug administration [see (11–14)].

In addition, the use of mixed cultures containing combinations of wild-type or abnormal, (i.e., genetically modified), neural cells were of great help to explore basic neuropathological mechanisms. Data obtainable from conventional two-dimensional cultures include aspects such as of neurite outgrowth, synaptogenesis, the release of neurotransmisors, being also useful to design protocols that direct differentiation of stem cell towards neuron and glia subtypes (15, 16). A major weakness of two-dimensional cultures (2D-cultures) came from their impossibility to replicate the complex architecture of neurons and glia in distinct brain regions that is of critical importance for function (17).

Attempts to develop three-dimensional cultures to study the structure and function of specific neural circuits, employed substrate scaffolds that support the formation of neural networks (18). In the last decade, advances in tissue bioengineering generated efficient 3D multicellular culture systems, termed “organoids” where cells growing in matrix substrates are able to self-organizing and re-capitulate, quite accurately, functional and structural development of the adult organs [reviewed by Goldrick et al. (19)]. Many methodology variations to the basic organoid technology has been introduce in the last years to adapt the assay to unravel specific questions (20). Among these variations are the formation of 3D cultures free of matrix scaffold, termed “spheroids,” or the combination of organoids obtained from distinct cell sources to explore interactions involving distinct cell types, that has been termed “assembloids” (21, 22). Overall, organoids provided a great methodological advance to study the bases of multiple human pathologies including neuropsychiatric disorders and, most important, to test the effects of different treatments in a personalized fashion [see (11, 12, 14)]. The progress achieved in the organoid technology in the last years has open the possibility to employ brain organoids in the next future, as biological chips for artificial intelligence (23, 24). The term of “organoid intelligence” has been proposed for this potential application (24).

First approaches in the design of three-dimensional cell cultures have been carried out using stem cells obtained largely from experimental animals. However, in the first decade of this century, the contribution of the Japanese Nobel awarded Shinya Yamanaka and other research groups in cell reprogramming has led to a revolution in the application of organoids to the study of human pathology. Takahashi and Yamanaka (25) managed to generate pluripotent stem cells by transfecting fibroblasts obtained from the skin of adult subjects with a cocktail of 4 genes, which encode for transcription factors. These cells were called “induced pluripotent stem cells” (iPSCs) and grown under appropriate conditions are capable of differentiating into all cell lines, including specific neuron subtypes and glia (26). The procedures to obtain IPSCs have been implemented in subsequent studies, allowing the use of different cells from adult tissues as a source to obtain iPSCs (27).

Since the studies by Lancaster et al. (28), it has been found that brain organoids replicate the establishment of interneuronal connections and the production of neurotransmitters. In addition, it has been verified that according to culture protocols (29) brain organoids can be designed to replicate the structural and neurochemical characteristics of specific regions of the CNS [see (7, 30, 31)], including diencephalon (32), brain stem (33), cerebellum (34), spinal cord (35, 36) and even to develop models of the cerebral cortex (37, 38). Furthermore, the combination of optogenetic techniques transfecting neural progenitors with genes that encode markers that are stimulated by light, allows highly sophisticated functional studies to be carried out (39).

Despite the extraordinary utility of organoids for the study of human brain development and pathology (40), they show weakness that need to be taken into account for appropriate modeling human diseases. From the technical point of view, a major shortcoming of current organoid methods is the lack of blood vessels in the culture. Due to insufficient surface diffusion, the interior of the organoid is under hypoxia resulting in central cell death. This causes slow tissue growth and developmental variability among distinct samples. Numerous efforts have been done to design procedures that ameliorate tissue nutrition. A relatively simple protocol is to slice organoids in thinner samples to bypass the diffusion limit and prevent cell death over long-term cultures. This method sustains development and neurogenesis in the organoid allowing the study of late stage cortical development (37).

A most promising modification of the tridimensional culture assays of special interest for drug screening, is the so-called “organ-on-a-chip” (41). In this assay, cells are cultured in micro-channels subjected to controlled fluid flow within a microfluidic device that is provided with biosensors to monitor biomarkers secreted by the organoids (42, 43). In addition to detect modifications induced by selected drug treatments, this assay enables to explore interactions between distinct organoids growing within channels interconnected together (“multiorgans microdevice”) or to generate a network of microvessels by adding growing vascular progenitors in connection with the organoid (44). Over-all, the organ-on-chip technology is a fast-moving field of research, and we could anticipate, that distinct types of these organ chip models will be manufactured and standardized in the next future [see (42, 45)].

In clinical medicine, the use of non-neural organoids offers the possibility of being used as a personalized test for therapeutic planning of tumor pathology and degenerative diseases, and the application of neural tissue organoids to the study of psychiatric disorders is promising. Since iPSCs are obtained from patients, the differentiated components in the organoid share their alterations, including genetic abnormalities. In addition, the response to drug administration could replicate, at least in part, that caused if it were administered to the patient.

There are however major limitations of organoid technology when applied to gain clinical insights of psychiatric disorders. The first one is the inability of organoids for modeling cognitive and behavioral symptoms that are core features of psychiatric disorders. In some way, this is the same that happens in animal models. Another major limitation refers to the importance of environmental biopsychosocial factors such as life experiences or substance abuse in the evolution of psychiatric disorders. While some environmental factors such as drug abuse could be tested in the organoid assay, most of them are out of the organoid resolution. A further limitation of organoid studies came from the implication of different brain regions in the pathophysiology of psychiatric diseases. However, different strategies have been proposed to solve, at least in part, these limitations. As mentioned above, “assembloids” and/or organ-on-chip models (46) has been developed for this purpose. In addition, it is now possible investigating the functional effect of organoid implantation in the brain of host experimental animals. This is a novel aspect in the experimental use of brain organoids, which combines the formation of human organoids with animal experimentation. Models of implantation of mature organoids in the brain of adult or newborn experimental animals are being developed (47). Organoids have been shown to integrate into the cerebral cortex of host animals and have a specific influence on functional aspects of the selected brain area (48, 49). At present, there is only tentative data on the possible relevance of this experimental approach. However, it should be mentioned that within the field of neurology, neurological deficits due to traumatic cortical lesions in mice have been alleviated by implanting human organoids in the injured area (50). These results have raised ethical concerns due to the risk that chimeric animals may experience a certain degree of humanization that generates an increased perception of suffering (51, 52).

In summary, the use of brain organoids from psychiatric patients allows at least the following data to be obtained:

1. Detecting functional and structural alterations of nervous organs complementary to those obtained by imaging studies and in autopsy samples.
2. Exploring the effect of new drugs on the alterations present in organoids.
3. Verifying, in a personalized fashion, the effect and efficacy of the different possible treatments for the patient’s disorder (53).
4. Investigating the functional effect of their implantation in the brain of host experimental animals.

In this essay, we gather data obtained through the use of brain organoids regarding three highly prevalent pathologies in psychiatric clinic, including schizophrenia, major depression disorder, and bipolar disorder.

### Schizophrenia

The elaboration of brain organoids through the use of iPSCs from schizophrenic patients has confirmed that it is a mental disorder associated with the development of the CNS, as well as confirming the alterations detected in postmortem studies and improving our knowledge about them (54). The most notable alterations of these organoids deal with neuronal development that included impaired differentiation of dopaminergic cells and lack of maturation of glutamatergic cells (55). From the cellular point of view, neurons with less dendritic branching and reduced synaptic connectivity are formed, and migration of neuroblasts within the tissue is deficient (56, 57). Functionally, electrophysiological alterations have been described due to abnormalities of Na^+^ channels, increased GABA-ergic neurotransmission (58) that generate imbalance between activating and inhibitory signaling (59, 60), and mitochondrial alterations accompanied by increased oxidative stress (61, 62). The later has been proposed to be a central feature of SCZD since transfer of normal mitochondria to SCZD-iPSCs cells improved differentiation of glutamatergic neurons and, *in vivo* similar treatment rescued attentional deficits in a rodent model of schizophrenia (63).

Consistent with the morphological and functional alterations, differences in the expression of a high number of genes and non-coding micro RNAs (mi-RNAs) with respect to controls have been also detected (56, 64). The panel of regulated genes includes components of the Wnt and cAMP signaling pathways (56), and regulation of the FGFR1 receptor (65, 66). Members of these signaling pathways play important roles in basic aspects of neural development and differentiation in the embryo. Other characteristic molecular alterations are the overexpression of the nuclear protein called disrupted in schizophrenia 1 (DIC 1); the reduction of the synaptic protein PSD95, that is a marker of excitatory synapses (67), and the mitotic arrest defient-1 gene (MAD1), that regulates neuronal migration (68). It is important to note that those proteins also regulate basic aspects of CNS development and neurotransmission.

Remarkably, not all the alterations have been observed in all the patients analyzed (67). Some of the alterations, such as MAD1 deficiency, or the increase expression of DISC 1, have been found also in other mental disorders such as major depression and bipolar disorder (69). We do not know if there is a common causal factor shared by these alterations, but it is known that major depression has a high incidence among schizophrenic patients (70).

Given that there are genetic variations between patients, the existence of different causal factors which converge in basic alterations of neural development, giving rise to a similar phenotype has been suggested (71). The heat shock-induced transcription factor, HSF1, has been identified as a protective factor in response to stress signals during embryonic development of the cerebral cortex whose regulation is deficient in iPSCs from schizophrenics (72). This fact would make the developing CNS of schizophrenics more vulnerable to different kinds of damaging agents (alcohol, hypoxia, convulsive pathology of the mother, etc.). In this same sense, it has been seen that organoids of schizophrenic patients are particularly vulnerable to exposure to the cytokine TNF alpha (tumor necrosis factor) generated by immune cells, suggesting that environmental factors, including maternal immune activation due to infections during pregnancy, can act as a trigger in fetuses with a genetic background of SCZD (73).

Concerning the evaluation of the therapeutic efficacy of drugs using brain organoids, it has been observed that treatments with the antipsychotic loxapine improves the connectivity of neurons by increasing the expression of glutamate receptors and reducing the deficiency in the expression of members of the WNT family (56).

### Major depression disorder

Major depression disorder (MDD) is a highly prevalent disorder in western countries, but its molecular bases are not yet fully established (74). Genetic epidemiological studies support a genetic basis with multiple subtype variations for this disorder (75). Clinical and pharmacologic studies support disruption of serotonergic neurotransmission as a central factor causal of the disorder and selective serotonin reuptake inhibitors (SSRIs) are considered first choice treatment. Numerous efforts were paid to design culture conditions to generate serotoninergic neuron from human iPSCs to explore alterations in MDD patients (76). However, the high number of patients resistant to SSRIs treatment (77), directed brain organoid-based research to elucidate the bases of resistance in order to unravel the pathophysiology of the disorder and to identify effective treatments.

When iPSCs from treatment-resistant patients have been studied, intensification of the response to serotonergic stimuli was detected in forebrain neurons associated with hyperactivity of excitatory serotonergic receptors (5-HT2A and 5-HT7) that did not appear in organoids from subjects normal or from MDD patients responding to SSRIs (78). This functional difference in treatment-resistant patients is associated with structural changes in serotonergic neurons, including increased growth of neuronal cell extensions and negative regulation of protocadherin alpha 6 and 8 genes involved in cell adhesion (79). An important aspect is that experimental silencing of protocadherin genes in organoids from healthy subjects replicated the branching expansion of serotonergic neurons detected in the MDDs of patients resistant to SSRIs, and protocadherin-KO mice display apparently depressive behaviors (80). Overall, studies suggest an important role for protocadherins in resistance to SSRI treatments.

In recent years, the glutamate N-methyl-d-aspartate (NMDA) receptor antagonist, Ketamine, has been used as a promising antidepressant with a very rapid (hours) and sustained response over time (more than 1 week) despite the fact that its half-life is very short (2 h). Using a brain organoid model, Cavalleri et al. (81) have observed that after the administration of ketamine there is an increase in the size of the soma and in the pattern of dendritic branching of dopaminergic neurons. These rapid-induced structural changes (6 h) are maintained for days, which could explain the sustainability of the treatment effects over time (82). On the other hand, the effect of ketamine was inhibited by adding rapamycin, which is a specific inhibitor of the receptor called mTOR involved in anabolic processes, and also by inhibitors of brain-derived neural growth factor, BDNF (81).

An alternative approach to the analysis of the mechanisms of action of antidepressants on MDD patients using brain organoids is the application of this technology to detect adverse effects on the embryo and fetuses of antidepressant drug treatments administered to pregnant women (83). Using this approach, Zohng et al. (84) have verified that treatments with 60 ng/mL of paroxetine decrease dendritic density and the population of oligodendrocytes in brain organoids of healthy subjects.

## Bipolar disorder

Bipolar disorder (BD) is a chronic psychiatric condition characterized by severe swings in mood, alternating periods of major depression and manic or hypomanic periods. The familial distribution, as well as the incidence between twin brothers, has revealed the hereditary profile of this pathology.

The use of brain organoids and iPSCs in the study of this pathology has focused on two fundamental aspects of the alteration: the characterization of the molecular bases involved in the pathogenesis; and in the analysis of the effect and mechanism of action of the drugs used to stabilize mood [lithium, valproic acid, lamotrigine (85)].

Although the samples analyzed are limited, and the results are often heterogeneous, modern genetic studies using iPSCs from families or individuals with BP detected a very large number of genes regulated differently from healthy controls, which often appear also altered in other psychiatric disorders such as MDD or SCZD (86, 87).

The organoids derived from BD patients develop a smaller size, have fewer neurons, and form less excitable and less complex networks than the controls (85). In these organoids, genes that code for membrane receptors and ion channels appear over-expressed, and Ca^++^ signaling is significantly disturbed (88), in addition to regulating a very large number of genes involved in maturation and neuronal plasticity. Regulated genes include members of the Wnt signaling pathway and other genes that appear also modified in organoids or in postmortem samples derived from SCZD patients (87, 89).

Mood stabilizers, especially lithium, are the treatment of choice for BD to which most patients respond, and it has been considered that clarifying their mechanism of action could provide relevant information to characterize the pathogenesis of the disease. Consistent with this interpretation, lithium pretreatment of BD organoids has been observed to modify Ca^++^ fluxes, and the expression of genes that confer topographic identity to telencephalic neurons during development (88). In addition, prolonged lithium treatments are associated with transcriptional regulation of more than 100 genes, and functionally attenuate the loss of excitability in BD organoids while having opposite effects in organoids from healthy control subjects (85). One significant aspect is that the response of iPSCs to mood stabilizers correlates with the response to treatment of the BP patients from whom they are derived (85, 90).

Preliminary studies using rat cerebellar neurons exposed to mood stabilizers (lithium and valproic acid) and subjected to glutamate excitotoxicity have shown that mood stabilizers have a neuroprotective effect mediated by the regulation of microRNAs [miR-34a, miR-147b, miR-182, miR-222, miR-495, and miR-690 (91)]. Out of all these microRNAs, miR-34a is overexpressed in organoids derived from BD patients and its experimental overexpression in organoids from healthy subjects alters neuronal morphology, represses their differentiation and reduces the expression of synaptic proteins (92). In a complementary fashion, silencing miR-34a promotes the expansion of dendritic arborization. Based on these effects, it has been proposed that this micro-RNA could constitute a central element on which different BD-inducing agents act to trigger this psychiatric disorder (92). MicroRNAs are small units of RNA that do not directly code for protein formation but rather modify the synthesis of specific target proteins by repressing the translation of the corresponding messenger RNA. Several factors regulated by miR-34a have been identified that support the idea of a critical position of this micro-RNA in the molecular cascade involved in the pathogenesis of BP. One of these targets is the cellular cytoskeleton regulatory phosphoprotein, called “collapsin response mediator protein-2” (CRMP2) (93). CRMP2 is involved in the formation of dendritic spines.

The effects of brain organoid exposure to valproic acid are of particular clinical interest because its administration during pregnancy has been associated with a high incidence of autism spectrum syndrome in offsprings. In forebrain organoids, valproic acid, at high doses, has inhibitory effects on growth and neurogenesis, recapitulating the teratogenic effect of its administration during pregnancy (94).

Beside to the aforementioned alterations in neurons, BD organoids show alterations in astrocytes that cause less functional support for neurons and a reduction in their excitability. This effect is associated with a high production of interleukin-6 (IL-6) (95). IL6 proinflammatory signaling has no effect on iPSCs from healthy subjects, and does not appear to be a specific aspect of BD as it is also observed in iPSCs from schizophrenic patients (96). According to this last study, the action of IL6 is carried out on microglial cells and is consistent with the role of maternal inflammatory processes as a trigger for both psychiatric conditions (96).

## Conclusions and prospects

Brain organoids emerge as a new methodological approach for the study of the nervous system that in combination with animal experimentation and large-scale genome-wide studies might provide in the next years a great advance in understanding the pathophysiology of psychiatric disorders providing also insights on new therapeutic approaches for those disorders.

From the numerous data obtained so far by employing iPSCs gathered in this essay we would emphasize four major conclusions: (i) that the origin of these pathologies takes place in the stages of embryonic development; (ii) the existence of shared molecular pathogenic aspects among patients of the three distinct disorders; (iii) the occurrence of molecular differences between patients bearing the same disorder; and, (iv) that functional alterations can be activated or aggravated by environmental signals in patients bearing genetic risk for these disorders.

In recent years, the abundance of shared symptomatology among psychiatric patients with distinct diagnosis together with the occurrence of considerable overlapping patterns of gene alterations in distinct mental disorders has led to a re-evaluation of the traditional categorical diagnostic classification of psychiatric nosology (DSM and ICD) for the advance of psychiatry (97). There is a growing consensus that a “*transdiagnostic”* approach focused in the analysis of common underlying mechanisms involved in multiple psychiatric disorders may be useful for a better understanding psychiatric disorders (98). Organoids can be used to investigate common mechanisms such as synaptic dysfunction, or neurogenesis deficits that may underlie multiple psychiatric conditions providing new criteria complementary to the information employed in transdiagnostic psychiatry (99). In this context, organoids can also serve as models for testing potential therapeutic interventions, which target these shared mechanisms rather than focusing on specific diagnostic.

## Author contributions

The author confirms being the sole contributor of this work and has approved it for publication.

## Conflict of interest

The author declares that the research was conducted in the absence of any commercial or financial relationships that could be construed as a potential conflict of interest.

## Publisher’s note

All claims expressed in this article are solely those of the authors and do not necessarily represent those of their affiliated organizations, or those of the publisher, the editors and the reviewers. Any product that may be evaluated in this article, or claim that may be made by its manufacturer, is not guaranteed or endorsed by the publisher.
